# [1-(4-Hydroxy­phen­yl)-1*H*-tetra­zol-5-ylsulfan­yl]acetic acid

**DOI:** 10.1107/S1600536808037136

**Published:** 2008-11-26

**Authors:** Cai-Hong Zhan, Shu-Guang Zhang, Yun-Long Feng

**Affiliations:** a Zhejiang Key Laboratory for Reactive Chemistry on Solid Surfaces, Institute of Physical Chemistry, Zhejiang Normal University, Jinhua, Zhejiang 321004, People’s Republic of China

## Abstract

The title compound, C_9_H_8_N_4_O_3_S, shows a layer structure constructed from inter­molecular O—H⋯O and O—H⋯N hydrogen bonds. Inter­atomic distances suggest that extensive, but not uniform, π-electron delocalization is present in the tetra­zole rings and extends over the exocyclic C—S bond.

## Related literature

For related literature on tetra­zol-5-thione and its derivatives, see: Cea-Olivares *et al.* (1997 ▶); Kim *et al.* (2003 ▶).
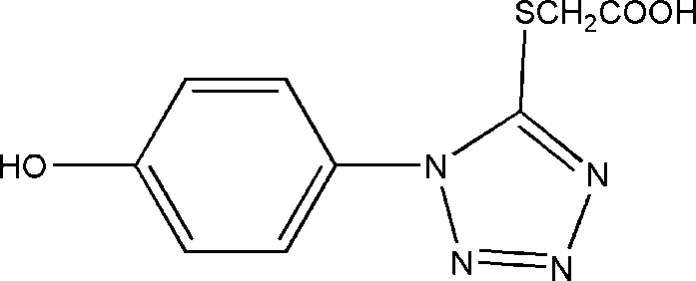


## Experimental

### 

#### Crystal data

C_9_H_8_N_4_O_3_S
*M* *_r_* = 252.25
Orthorhombic, 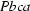

*a* = 14.407 (3) Å
*b* = 7.3365 (16) Å
*c* = 21.107 (5) Å
*V* = 2231.0 (9) Å^3^
*Z* = 8
Mo *K*α radiation
μ = 0.29 mm^−1^
*T* = 293 K
0.28 × 0.16 × 0.10 mm

#### Data collection

Bruker APEXII area-detector diffractometer
Absorption correction: multi-scan (*SADABS*; Sheldrick, 1996 ▶) *T*_min_ = 0.95, *T*_max_ = 0.97
18595 measured reflections
2550 independent reflections
1834 reflections with *I* > 2σ(*I*)
*R*_int_ = 0.099

#### Refinement

*R*[*F* ^2^ > 2σ(*F* ^2^)] = 0.047
*wR*(*F* ^2^) = 0.106
*S* = 1.06
2550 reflections
160 parameters
2 restraints
H atoms treated by a mixture of independent and constrained refinement
Δρ_max_ = 0.19 e Å^−3^
Δρ_min_ = −0.21 e Å^−3^

### 

Data collection: *APEX2* (Bruker, 2002 ▶); cell refinement: *SAINT* (Bruker, 2002 ▶); data reduction: *SAINT*; program(s) used to solve structure: *SHELXS97* (Sheldrick, 2008 ▶); program(s) used to refine structure: *SHELXL97* (Sheldrick, 2008 ▶); molecular graphics: *SHELXTL* (Sheldrick, 2008 ▶); software used to prepare material for publication: *SHELXL97*.

## Supplementary Material

Crystal structure: contains datablocks I, global. DOI: 10.1107/S1600536808037136/at2673sup1.cif

Structure factors: contains datablocks I. DOI: 10.1107/S1600536808037136/at2673Isup2.hkl

Additional supplementary materials:  crystallographic information; 3D view; checkCIF report

## Figures and Tables

**Table 1:** Hydrogen-bond geometry (Å, °)

*D*—H⋯*A*	*D*—H	H⋯*A*	*D*⋯*A*	*D*—H⋯*A*
O1—H1⋯O3^i^	0.835 (17)	1.953 (17)	2.787 (3)	177 (3)
O2—H2⋯N4^ii^	0.834 (17)	1.866 (17)	2.699 (3)	176 (3)
O2—H2⋯N3^ii^	0.834 (17)	2.60 (2)	3.369 (3)	154 (3)

Symmetry codes: (i) 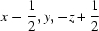; (ii) 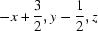.

**Fig. 1.**
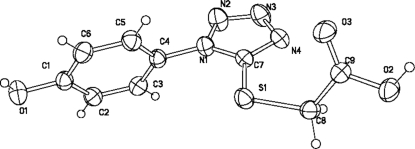
The molecular structure of the title compound, showing 30% probability displacement ellipsoids

## supplementary crystallographic information

### Comment

Tetrazol-5-thione and its derivatives are interesting ligands from a structural point of view since they can display a wide range of coordination patterns with metal ions. Due to a variety of potential coordination sites, they can act as monodentate (–S or –N) or bidentate (–N, N or –N, S) ligands, forming polymers or interacting with metal ions (Cea-Olivares *et al.*, 1997; Kim *et al.*, 2003).

As shown in Fig.1, the bond lengths within the tetrazole ring exhibit the expected pattern of four long bonds (C7—N1, C7—N4, N3—N4 and N1—N2) together with a short one (N2—N3). In detail, C7—N1 [1.340 (2) Å] and C7—N4 [1.324 (2) Å] are typical for carbon-nitrogen single bonds from 1.336 Å to 1.420 Å, while N3—N4 [1.365 (2) Å] and N1—N2 [1.358 (2) Å] are between the single and double bonds. And the bond distance N2—N3 of 1.283 (2) Å is similar to that of a double bond of 1.25 Å. The bond length of S1—C7 [1.723 (2) Å9 also falls between the double and single bonds. All these interatomic distances suggest that extensive but not uniform π electron delocalization is present in the tetrazole rings and extends over the exocyclic C—S bond.

### Experimental

To an aqueous solution of 1-(4-hydroxyphenyl)-5-thiotetrazole (1.940 g, 10.0 mmol) and NaOH (0.80 g, 20.0 mmol) were sequentially added the aqueous solution of chloroactic acid (2.835 g, 30.0 mmol) and NaOH (1.400 g, 35.0 mmol). After stirring for 4 h at 353 K under nitrogen atmosphere, the mixture was cooled to room temperature slowly. Adjusted the pH to 2 by adding 1.0 mol/*L* HCl, the white deposit appeared rapidly. The solids were filtered and washed with water. The single crystals suitable for X-ray diffraction were obtained by the re-crystallization of sieved solid in the ethanol.

### Refinement

The H atoms bonded to C atoms were positioned geometrically and treated as riding, [aromatic C—H = 0.93 Å and aliphatic C—H = 0.97 Å, *U*_iso_(H) = 1.2*U*_eq_(C)]. The H atoms bonded to O atoms were located in a difference Fourier map and refined isotropically.

### Crystal data

**Table:**

C_9_H_8_N_4_O_3_S	*F*_000_ = 1040
*M**_r_* = 252.25	*D*_x_ = 1.502 Mg m^−^^3^
Orthorhombic, *P**b**c**a*	Mo *K*α radiation λ = 0.71073 Å
Hall symbol: -P 2ac 2ab	Cell parameters from 2531 reflections
*a* = 14.407 (3) Å	θ = 2.4–27.5º
*b* = 7.3365 (16) Å	µ = 0.29 mm^−^^1^
*c* = 21.107 (5) Å	*T* = 293 K
*V* = 2231.0 (9) Å^3^	Block, colourless
*Z* = 8	0.28 × 0.16 × 0.10 mm

### Data collection

**Table:**

Bruker APEXII area-detector diffractometer	2550 independent reflections
Radiation source: fine-focus sealed tube	1834 reflections with *I* > 2σ(*I*)
Monochromator: graphite	*R*_int_ = 0.099
*T* = 293 K	θ_max_ = 27.5º
ω scans	θ_min_ = 2.4º
Absorption correction: multi-scan(SADABS; Sheldrick, 1996)	*h* = −18→18
*T*_min_ = 0.95, *T*_max_ = 0.97	*k* = −9→9
18595 measured reflections	*l* = −27→27

### Refinement

**Table:**

Refinement on *F*^2^	Secondary atom site location: difference Fourier map
Least-squares matrix: full	Hydrogen site location: inferred from neighbouring sites
*R*[*F*^2^ > 2σ(*F*^2^)] = 0.047	H atoms treated by a mixture of independent and constrained refinement
*wR*(*F*^2^) = 0.106	*w* = 1/[σ^2^(*F*_o_^2^) + (0.0144*P*)^2^ + 1.7635*P*] where *P* = (*F*_o_^2^ + 2*F*_c_^2^)/3
*S* = 1.06	(Δ/σ)_max_ = 0.001
2550 reflections	Δρ_max_ = 0.19 e Å^−^^3^
160 parameters	Δρ_min_ = −0.21 e Å^−^^3^
2 restraints	Extinction correction: none
Primary atom site location: structure-invariant direct methods	

### Special details

**Table:**

Geometry. All e.s.d.'s (except the e.s.d. in the dihedral angle between two l.s. planes) are estimated using the full covariance matrix. The cell e.s.d.'s are taken into account individually in the estimation of e.s.d.'s in distances, angles and torsion angles; correlations between e.s.d.'s in cell parameters are only used when they are defined by crystal symmetry. An approximate (isotropic) treatment of cell e.s.d.'s is used for estimating e.s.d.'s involving l.s. planes.
Refinement. Refinement of *F*^2^ against ALL reflections. The weighted *R*-factor *wR* and goodness of fit *S* are based on *F*^2^, conventional *R*-factors *R* are based on *F*, with *F* set to zero for negative *F*^2^. The threshold expression of *F*^2^ > σ(*F*^2^) is used only for calculating *R*-factors(gt) *etc*. and is not relevant to the choice of reflections for refinement. *R*-factors based on *F*^2^ are statistically about twice as large as those based on *F*, and *R*- factors based on ALL data will be even larger.

### Fractional atomic coordinates and isotropic or equivalent isotropic displacement parameters (Å^2^)

**Table:**

	*x*	*y*	*z*	*U*_iso_*/*U*_eq_	
S1	0.51454 (4)	0.15070 (9)	0.09961 (3)	0.05074 (19)	
O1	0.15861 (13)	0.3256 (3)	0.28120 (9)	0.0610 (5)	
H1	0.177 (2)	0.270 (4)	0.3133 (11)	0.073*	
O2	0.75012 (12)	0.0102 (3)	0.01914 (8)	0.0551 (5)	
H2	0.8048 (13)	0.010 (4)	0.0322 (13)	0.066*	
O3	0.72494 (12)	0.1471 (3)	0.11139 (8)	0.0645 (5)	
N1	0.45507 (13)	0.4915 (3)	0.12461 (10)	0.0468 (5)	
N2	0.47350 (16)	0.6679 (3)	0.10961 (12)	0.0637 (6)	
N3	0.54403 (16)	0.6653 (3)	0.07237 (12)	0.0619 (6)	
N4	0.57366 (13)	0.4912 (3)	0.06208 (10)	0.0480 (5)	
C1	0.23393 (16)	0.3617 (3)	0.24421 (11)	0.0441 (5)	
C2	0.21686 (16)	0.4284 (3)	0.18436 (12)	0.0470 (6)	
H2A	0.1561	0.4457	0.1708	0.056*	
C3	0.28941 (16)	0.4695 (3)	0.14462 (12)	0.0478 (6)	
H3A	0.2783	0.5152	0.1042	0.057*	
C4	0.37920 (15)	0.4418 (3)	0.16560 (11)	0.0436 (5)	
C5	0.39734 (17)	0.3749 (4)	0.22511 (12)	0.0509 (6)	
H5A	0.4582	0.3576	0.2386	0.061*	
C6	0.32408 (17)	0.3336 (4)	0.26463 (12)	0.0509 (6)	
H6A	0.3352	0.2870	0.3049	0.061*	
C7	0.51693 (14)	0.3855 (3)	0.09473 (11)	0.0402 (5)	
C8	0.59818 (15)	0.0925 (3)	0.03998 (12)	0.0465 (6)	
H8A	0.5826	−0.0265	0.0231	0.056*	
H8B	0.5931	0.1797	0.0056	0.056*	
C9	0.69718 (16)	0.0887 (3)	0.06206 (11)	0.0429 (5)	

### Atomic displacement parameters (Å^2^)

**Table:**

	*U*^11^	*U*^22^	*U*^33^	*U*^12^	*U*^13^	*U*^23^
S1	0.0418 (3)	0.0418 (3)	0.0686 (4)	−0.0030 (3)	0.0098 (3)	−0.0001 (3)
O1	0.0472 (10)	0.0753 (14)	0.0607 (11)	0.0032 (9)	0.0128 (9)	0.0151 (10)
O2	0.0389 (8)	0.0757 (13)	0.0508 (10)	0.0116 (9)	−0.0034 (8)	−0.0121 (9)
O3	0.0490 (11)	0.0903 (15)	0.0542 (11)	0.0078 (10)	−0.0099 (8)	−0.0217 (11)
N1	0.0421 (11)	0.0421 (12)	0.0563 (12)	0.0009 (9)	0.0080 (9)	0.0042 (10)
N2	0.0611 (14)	0.0407 (12)	0.0894 (17)	0.0025 (11)	0.0171 (13)	0.0099 (12)
N3	0.0520 (13)	0.0486 (13)	0.0850 (16)	−0.0031 (11)	0.0140 (12)	0.0113 (12)
N4	0.0375 (10)	0.0481 (12)	0.0584 (12)	−0.0027 (9)	0.0030 (9)	0.0048 (10)
C1	0.0426 (13)	0.0412 (13)	0.0484 (13)	0.0017 (10)	0.0062 (10)	−0.0012 (11)
C2	0.0389 (12)	0.0491 (14)	0.0529 (14)	0.0016 (11)	−0.0015 (11)	0.0025 (12)
C3	0.0486 (14)	0.0487 (15)	0.0460 (13)	0.0035 (11)	−0.0012 (11)	0.0050 (11)
C4	0.0414 (12)	0.0392 (12)	0.0502 (14)	0.0007 (10)	0.0080 (11)	0.0005 (11)
C5	0.0391 (12)	0.0588 (16)	0.0547 (15)	0.0029 (11)	−0.0014 (11)	0.0016 (12)
C6	0.0515 (14)	0.0555 (15)	0.0456 (14)	0.0018 (12)	0.0002 (11)	0.0041 (12)
C7	0.0319 (11)	0.0428 (13)	0.0458 (12)	−0.0020 (9)	0.0000 (10)	0.0000 (10)
C8	0.0394 (12)	0.0443 (14)	0.0558 (14)	0.0005 (10)	−0.0043 (11)	−0.0080 (11)
C9	0.0408 (12)	0.0445 (13)	0.0435 (13)	0.0040 (10)	−0.0030 (11)	0.0003 (11)

### Geometric parameters (Å, °)

**Table:**

S1—C7	1.726 (2)	C1—C2	1.377 (3)
S1—C8	1.794 (2)	C1—C6	1.384 (3)
O1—C1	1.363 (3)	C2—C3	1.374 (3)
O1—H1	0.835 (17)	C2—H2A	0.9300
O2—C9	1.317 (3)	C3—C4	1.382 (3)
O2—H2	0.834 (17)	C3—H3A	0.9300
O3—C9	1.195 (3)	C4—C5	1.374 (3)
N1—C7	1.341 (3)	C5—C6	1.379 (3)
N1—N2	1.359 (3)	C5—H5A	0.9300
N1—C4	1.441 (3)	C6—H6A	0.9300
N2—N3	1.285 (3)	C8—C9	1.501 (3)
N3—N4	1.364 (3)	C8—H8A	0.9700
N4—C7	1.321 (3)	C8—H8B	0.9700
			
C7—S1—C8	100.49 (11)	C3—C4—N1	118.7 (2)
C1—O1—H1	108 (2)	C4—C5—C6	119.1 (2)
C9—O2—H2	109 (2)	C4—C5—H5A	120.5
C7—N1—N2	108.24 (19)	C6—C5—H5A	120.5
C7—N1—C4	129.8 (2)	C5—C6—C1	119.8 (2)
N2—N1—C4	121.94 (19)	C5—C6—H6A	120.1
N3—N2—N1	106.4 (2)	C1—C6—H6A	120.1
N2—N3—N4	111.0 (2)	N4—C7—N1	108.4 (2)
C7—N4—N3	105.86 (19)	N4—C7—S1	129.03 (18)
O1—C1—C2	116.9 (2)	N1—C7—S1	122.53 (17)
O1—C1—C6	122.7 (2)	C9—C8—S1	115.16 (17)
C2—C1—C6	120.4 (2)	C9—C8—H8A	108.5
C3—C2—C1	120.2 (2)	S1—C8—H8A	108.5
C3—C2—H2A	119.9	C9—C8—H8B	108.5
C1—C2—H2A	119.9	S1—C8—H8B	108.5
C2—C3—C4	119.0 (2)	H8A—C8—H8B	107.5
C2—C3—H3A	120.5	O3—C9—O2	124.2 (2)
C4—C3—H3A	120.5	O3—C9—C8	125.6 (2)
C5—C4—C3	121.6 (2)	O2—C9—C8	110.2 (2)
C5—C4—N1	119.7 (2)		
			
C7—N1—N2—N3	−0.2 (3)	C4—C5—C6—C1	−0.7 (4)
C4—N1—N2—N3	−179.5 (2)	O1—C1—C6—C5	−179.3 (2)
N1—N2—N3—N4	−0.2 (3)	C2—C1—C6—C5	0.9 (4)
N2—N3—N4—C7	0.5 (3)	N3—N4—C7—N1	−0.6 (3)
O1—C1—C2—C3	179.4 (2)	N3—N4—C7—S1	177.64 (19)
C6—C1—C2—C3	−0.7 (4)	N2—N1—C7—N4	0.5 (3)
C1—C2—C3—C4	0.4 (4)	C4—N1—C7—N4	179.7 (2)
C2—C3—C4—C5	−0.2 (4)	N2—N1—C7—S1	−177.85 (18)
C2—C3—C4—N1	−177.7 (2)	C4—N1—C7—S1	1.3 (3)
C7—N1—C4—C5	70.7 (3)	C8—S1—C7—N4	−7.9 (2)
N2—N1—C4—C5	−110.2 (3)	C8—S1—C7—N1	170.13 (19)
C7—N1—C4—C3	−111.7 (3)	C7—S1—C8—C9	86.2 (2)
N2—N1—C4—C3	67.4 (3)	S1—C8—C9—O3	−12.3 (4)
C3—C4—C5—C6	0.3 (4)	S1—C8—C9—O2	167.39 (17)
N1—C4—C5—C6	177.9 (2)		

### Hydrogen-bond geometry (Å, °)

**Table:**

*D*—H···*A*	*D*—H	H···*A*	*D*···*A*	*D*—H···*A*
O1—H1···O3^i^	0.835 (17)	1.953 (17)	2.787 (3)	177 (3)
O2—H2···N4^ii^	0.834 (17)	1.866 (17)	2.699 (3)	176 (3)
O2—H2···N3^ii^	0.834 (17)	2.60 (2)	3.369 (3)	154 (3)

Symmetry codes: (i) *x*−1/2, *y*, −*z*+1/2; (ii) −*x*+3/2, *y*−1/2, *z*.
